# Identifying early blood glucose trajectories in sepsis linked to distinct long-term outcomes: a K-means clustering study with external validation

**DOI:** 10.3389/fimmu.2025.1610519

**Published:** 2025-06-05

**Authors:** Huan Ma, Xiayan Qian, Xiaodong Song, Rongjie Jiang, Jialin Li, Fang Xiao, Ruoxu Dou, Xiangdong Guan, Ka Yin Lui, Shuhe Li, Changjie Cai

**Affiliations:** ^1^ Department of Critical Care Medicine, The First Affiliated Hospital of Sun Yat-Sen University, Guangzhou, Guangdong, China; ^2^ University of Exeter Medical School, University of Exeter, Exeter, United Kingdom

**Keywords:** sepsis, blood glucose, 1-year mortality, trajectory analysis, K-means clustering

## Abstract

**Background:**

Blood glucose (BG) dysregulation, including hyperglycemia, hypoglycemia and increased glycemic variability (GV), is common in septic patients and potentially associated with poor clinical outcomes. However, the prognostic value of early BG trajectories remains unclear. We intend to investigate the association between the early dynamic trajectory of BG and 1-year mortality among sepsis patients.

**Methods:**

This retrospective study comprises a derivation cohort of sepsis patients admitted to the First Affiliated Hospital of Sun Yat-sen University (FAH-SYSU) from January 2018 to December 2023, and an external validation cohort of 10,874 sepsis patients from the Medical Information Mart for Intensive Care (MIMIC) IV database. Distinct clusters were demarcated using K-means clustering based on the BG trajectory within the first 48 hours after ICU admission, while the optimal number of clusters was determined by a consensus of quantitative metrics and the elbow plot. Kaplan-Meier survival curves and multivariable Cox proportional hazards regression models were used to assess the association between these identified clusters and 1-year mortality.

**Results:**

Among 3,655 sepsis patients from the FAH-SYSU dataset, we identified 5 distinct clusters of BG trajectories, which were significantly associated with 1-year mortality risk. In the full Cox regression model, patients with “low-stable” and “moderate-stable” trajectories had the lowest 1-year mortality risk (*P* = 0.077). Conversely, patients with a “high-stable” trajectory (HR: 1.61, 95% CI: 1.35-1.92, *P* < 0.001) and those exhibiting unstable trends had significantly higher mortality risks (“high-decreasing”, HR: 1.38, 95% CI: 1.16-1.65, *P* < 0.001; “moderate-increasing”, HR: 1.37, 95% CI: 1.18-1.60, *P* < 0.001). External validation found consistent clusters with similar mortality trends. Restricted cubic spline analysis demonstrated a U-shaped association for mean glucose levels and a J-shaped relationship for GV linked to 1-year mortality risks, while an optimal glycemic range of 122 to 160 mg/dL and GV less than 0.18 indicated improved survival.

**Conclusion:**

Early BG trajectory patterns are independently associated with long-term mortality in sepsis patients. Incorporating dynamic BG measurements into clinical practice may improve risk stratification and guide individualized glucose management strategies.

## Background

Sepsis is a life-threatening organ dysfunction caused by a dysregulated host response to infection and remains one of the leading causes of mortality among intensive care units (ICU) patients (1). A key component of sepsis pathophysiology is a systemic inflammatory response, which involves the release of both pro- and anti-inflammatory cytokines, as well as counter-regulatory hormones (2). These metabolic alterations often result in hyperglycemia, a common complication in septic patients (3). Hyperglycemia triggers oxidative stress, mitochondrial dysfunction, cell death, tissue injury, and ultimately, organ failure (4–7).

Despite these findings, the optimal range of blood glucose (BG) in patients with sepsis remains controversial (6, 8, 9). While early studies suggested that strict glycemic control could improve outcomes, subsequent trials failed to demonstrate consistent benefits and even highlighted potential risks, including severe hypoglycemia (10–15). As a result, the Surviving Sepsis Campaign now recommends a more lenient glycemic target (<180 mg/dL) instead of the previously advocated tight control (<110 mg/dL) (8). Yet, the optimal glucose management strategy in sepsis remains a topic of debate, as some patients appear to benefit from tighter glucose control while others do not.

Beyond persistent hyperglycemia, glycemic variability (GV) has emerged as a significant concern in critically ill patients. GV refers to fluctuations in blood glucose levels over time, encompassing both hypoglycemic episodes and postprandial spikes (16). Studies have shown that increased GV is independently associated with poor prognosis and increased mortality in septic patients (17, 18). Given the association between GV and adverse outcomes, various methods have been proposed to quantify glucose fluctuations, including mean glucose concentration, mean absolute glucose (MAG) change, standard deviation (SD), and incidences of hypo- and hyperglycemia. However, the long-term prognostic impact of GV in sepsis patients, as well as the optimal range for these fluctuations, remains to be defined.

Recent research suggests that dynamic changes in blood glucose levels over time—rather than static glucose measurements—may provide more valuable prognostic insights (19–21). The concept of glucose level trajectories, which describes longitudinal changes in blood glucose concentrations, has been explored in some diseases like acute ischemic stroke. Li et al. showed that individuals with longitudinally elevated fasting glucose level trajectories had a higher risk of death even if they had normal glucose levels at baseline (19). Despite these findings, the prognostic relevance of early glucose trajectories specifically in sepsis has yet to be fully clarified.

Accordingly, our study aimed to develop and externally validate distinct clusters of BG trajectories based on two independent cohorts of sepsis patients using a machine learning technique (K-means clustering), and investigate the association between these diverse trajectories within the first 48 hours after ICU admission and 1-year mortality, thereby providing valuable insights into early glycemic patterns as prognostic indicators and informing personalized glycemic strategies in the ICU management.

## Methods

### Study design and population

We conducted a retrospective observational study utilizing data from two independent cohorts. The derivation cohort was collected from the First Affiliated Hospital of Sun Yat-sen University database (FAH-SYSU) (Guangzhou, China), comprising data from 10,029 ICU admissions between January 2018 and December 2023. Access to clinical data was approved by the Clinical Research Ethics Committee of the First Affiliated Hospital of Sun Yat-sen University (Institutional Review Board number: 2022-048). Additionally, the validation cohort was obtained from the Medical Information Mart for Intensive Care IV (MIMIC-IV) database, which contains a total of 299,712 ICU admissions at the Beth Israel Deaconess Medical Center (Boston, USA) between 2008 and 2019. One author (S. Li) is certified to get access to the database and was responsible for data extraction. As the data in the MIMIC-IV database were fully anonymized, the Institutional Review Board at the Beth Israel Deaconess Medical Center granted a waiver of informed consent (No.2001P001699).

Sepsis patients were diagnosed during ICU stay according to the third international consensus definition, that is, suspected infection accompanied by Sequential Organ Failure Assessment (SOFA) score 2 points higher than baseline (1). For patients with repetitive ICU admissions, only the first ICU admission was considered. Exclusion criteria included: age less than 18 years old, ICU length of stay less than 48 hours, less than 4 times of blood glucose measurements within the first 48 hours after ICU admission, and lack of follow-up information or unplanned discharge due to non-medical reasons. These criteria were applied to both cohorts.

### Data extraction

The following variables were extracted: (1) demographic information, for instance, age, gender, surgical status, Charlson Comorbidity Index (CCI), disease severity scores (Acute Physiologic Assessment and Chronic Health Evaluation [APACHE] II score and SOFA score), (2) laboratory results and vital signs on ICU admission, including hemoglobin, white blood cell (WBC) count, platelet count, creatinine, total bilirubin (TBIL), prothrombin time (PT), and procalcitonin (PCT), (3) blood glucose levels within 48 hours of ICU admission, (4) 1-year mortality, 30-day mortality, ICU length of stay (ICU-LOS), the need of organ support (vasopressors, mechanical ventilation, continuous renal replacement therapy [CRRT]). All data were extracted using Structured Query Language (SQL) queries via the pgAdmin4 (version 6.15) interface for PostgreSQL.

### Outcomes

The main outcome was all-cause 1-year mortality after ICU admission. Secondary outcomes included 30-day mortality, ICU mortality, ICU length of stay, vasopressor dosages, the administration of CRRT, ventilation duration, and doses of insulin.

### Exposure and clustering

The primary exposure in this study was blood glucose level trajectories within 48 hours after ICU admission. Specifically, we used the maximum blood glucose values in every 8-hour block, which were then standardized by subtracting the mean and dividing by the standard deviation, to form clusters with similar dynamic trends. In cases with missing glucose values, we imputed using the mean of each patient’s available measurements. Alternative methods (LOCF, NOCB, and MICE) showed consistent clustering and survival results, confirming the soundness of this approach.

Next, we utilized K-means clustering to group patients into distinct clusters, employing the Scikit-learn package (version 1.5.2). This study was conducted according to the CAIR checklist (**Appendix 1**) (22).To determine the optimal number of clusters (K), multiple metrics were examined, including the sum of squared errors (SSE), Silhouette Score, Calinski-Harabasz index, and Davies-Bouldin index (23–25). Besides, an elbow plot was generated to visualize the K and SSE, assisting in identifying a point where increasing K no longer significantly improved the fit. The final choice of K was based on a consensus among these measures and a qualitative inspection of the elbow plot. Each patient was then assigned to the resulting clusters, and subsequent analyses were conducted to compare clinical characteristics and outcomes across these groups.

Moreover, we performed an external validation using the MIMIC-IV database, ensuring the generalizability of the derived clusters. The same data preprocessing and clustering processes were replicated in this cohort.

**Figure 1:**
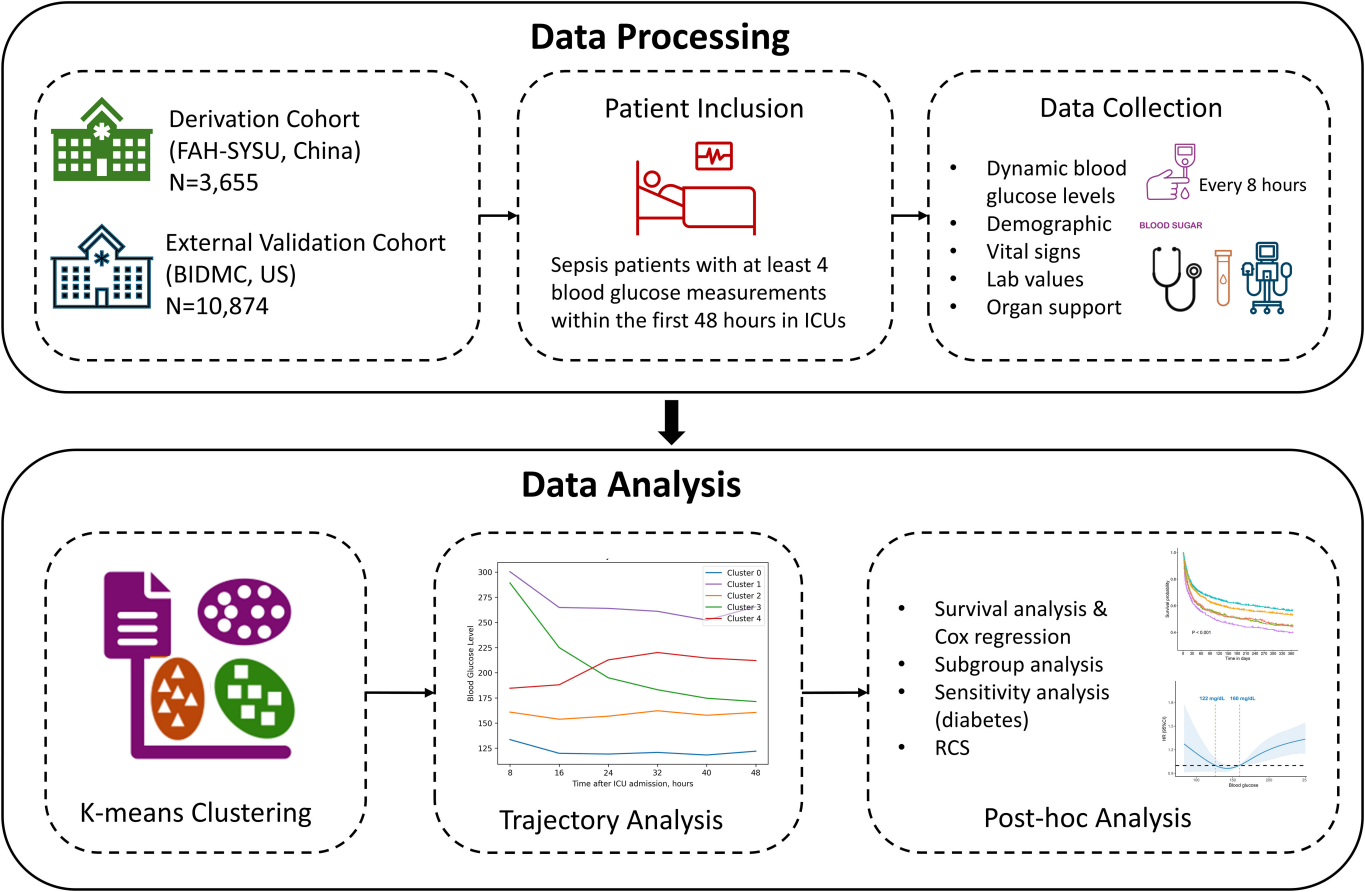
Schematic workflow of the study design.

To improve transparency and reproducibility, we incorporated a schematic workflow diagram (**Figure 1**) to visually summarize the study design and analytical pipeline. This diagram illustrates the key stages of the study: cohort selection and exclusion criteria, preprocessing of dynamic BG measurements, trajectory clustering using k-means, external validation in an independent cohort, and the subsequent outcome analyses including 1-year mortality comparisons across clusters. This visual representation helps clarify the sequence and rationale of the analytical steps.

### Statistical analyses

Continuous variables were reported as mean (standard deviation [SD]), while categorical variables were reported as counts with percentages. Continuous variables were compared with a one-way ANOVA test or the Kruskal-Wallis test based on the normality of candidate variables. Categorical variables were compared with either a chi-squared test or Fisher’s exact test when appropriate. Kaplan-Meier curves and log-rank tests were used to compare the 1-year survival outcomes across the groups. Additionally, we used both univariate and two multivariate Cox proportional hazards regression models (Model 1: adjusted for age and gender; Model 2: adjusted for age, gender, admission department, CCI, APACHE II, SOFA, creatinine, total bilirubin, hemoglobin, heart rate, mean arterial pressure, and urine output) to inspect the association between the formed clusters and 1-year survival risks.

To further validate the credibility of the identified clusters, subgroup analyses were performed stratifying by age (<=65 and > 65 years), gender, presence of septic shock, and surgical status, in order to evaluate the hazard ratios of 1-year morality within each subgroup. We also conducted a sensitivity analysis among patients with and without pre-existing diabetes to assess whether the clusters were associated with similar outcomes regardless of diabetes status. The significance of the interaction was assessed using the Wald test, and P values for interaction were reported. A P less than 0.05 was considered indicative of a statistically significant interaction. Lastly, restricted cubic spline (RCS) with four knots was adopted to visualize the potential non-linear relationships between two glycemic indexes (mean blood glucose levels, and the coefficients of variation [CV] within the 48 hours after ICU admission) and 1-year mortality. CV is an indicator for glucose variability in continuous glucose monitoring (26), calculated as the standard deviation of glucose values divided by the mean values in the same observation period.

Statistical significance was defined as a two-tailed *P* value of less than 0.05. All statistical analyses were conducted using R (version 4.2.2) or Python (version 3.12.2).

## Results

### Derivation cohort characteristics

A total of 4,608 patients diagnosed with sepsis were admitted to FAH-SYSU between January 2018 to December 2023. After screening, 953 patients were excluded, with 3,655 eligible patients included in our study. The process of participant enrollment is displayed in **Supplementary Figure S1A**. Among these patients, the mean age was 58.71 years (SD: 16.20), with 70.26% as male patients. The mean APACHE II score at admission was 20.39 (SD: 7.76), and 54.31% presented with septic shock (N=1,985), indicating high disease severity. The mean blood glucose level at admission was 164.82 mg/dL (SD: 75.01). There were 1,674 (45.80%) patients who died within 1 year after diagnosis with sepsis, as shown in **Table 1**.

**Table 1:** Demographic and clinical characteristics of sepsis patients stratifying by blood glucose trajectory clusters in the derivation cohort (N=3,655).

Variables	Overall n = 3655	Cluster 0 n = 1120	Cluster 1 n = 365	Cluster 2 n = 1157	Cluster 3 n = 393	Cluster 4 n = 620	*P* Value
Age, mean (SD), y	58.71 (16.20)	56.22 (17.74)	59.87 (15.70)	59.23 (15.22)	59.44 (15.15)	61.06 (15.44)	<0.001
Sex, n (%)							0.66
Female	1087 (29.74)	344 (30.71)	103 (28.22)	329 (28.44)	123 (31.30)	188 (30.32)	
Male	2568 (70.26)	776 (69.29)	262 (71.78)	828 (71.56)	270 (68.70)	432 (69.68)	
BMI, mean (SD)	22.37 (2.93)	22.01 (3.15)	22.55 (2.67)	22.45 (2.86)	22.62 (2.87)	22.59 (2.77)	<0.001
CCI, mean (SD)	3.48 (2.12)	3.30 (2.15)	3.26 (2.18)	3.61 (2.04)	3.58 (2.25)	3.64 (2.08)	<0.001
Admission department, n (%)							<0.001
Surgery	2053 (56.17)	637 (56.88)	167 (45.75)	702 (60.67)	222 (56.49)	325 (52.42)	
Non-surgery	221 (6.05)	60 (5.36)	20 (5.48)	66 (5.70)	26 (6.62)	49 (7.90)	
ICU or Emergency	939 (25.69)	259 (23.12)	150 (41.10)	250 (21.61)	104 (26.46)	176 (28.39)	
Other	442 (12.09)	164 (14.64)	28 (7.67)	139 (12.01)	41 (10.43)	70 (11.29)	
Surgical type, n (%)							<0.001
Gastrointestinal surgery	741 (20.27)	244 (21.79)	49 (13.42)	257 (22.21)	59 (15.01)	132 (21.29)	
Hepatobiliary surgery	720 (19.70)	166 (14.82)	70 (19.18)	266 (22.99)	90 (22.90)	128 (20.65)	
Vascular surgery	240 (6.57)	83 (7.41)	22 (6.03)	71 (6.14)	26 (6.62)	38 (6.13)	
Orthopedic surgery	207 (5.66)	85 (7.59)	21 (5.75)	56 (4.84)	23 (5.85)	22 (3.55)	
Other surgery	230 (6.29)	70 (6.25)	30 (8.22)	67 (5.79)	21 (5.34)	42 (6.77)	
Non-surgery	1517 (41.50)	472 (42.14)	173 (47.40)	440 (38.03)	174 (44.27)	258 (41.61)	
Severity score, mean (SD)							
APACHE II	20.39 (7.76)	18.63 (7.08)	23.21 (8.77)	19.81 (7.30)	22.06 (7.99)	21.90 (8.01)	<0.001
SOFA	7.56 (3.75)	7.07 (3.67)	7.74 (3.76)	7.65 (3.81)	7.99 (3.71)	7.89 (3.69)	<0.001
Laboratory result, mean (SD)							
BG on admission, mg/dL	164.82 (75.01)	121.57 (37.84)	269.52 (91.85)	144.01 (41.69)	249.93 (86.93)	166.18 (48.74)	<0.001
Mean BG in 48hours, mg/dL	171.49 (48.17)	122.31 (13.79)	268.06 (26.59)	158.60 (11.88)	206.64 (21.28)	205.27 (16.57)	<0.001
CV of BG in 48hours	0.20 (0.10)	0.17 (0.09)	0.22 (0.10)	0.17 (0.09)	0.28 (0.13)	0.20 (0.11)	<0.001
Prothrombin time, s	18.23 (7.75)	17.95 (6.49)	17.58 (6.65)	18.22 (6.73)	18.38 (6.87)	19.04 (11.68)	0.03
CRP, mg/L	124.88 (85.22)	117.78 (81.63)	141.87 (92.67)	122.54 (83.48)	124.71 (87.76)	132.28 (86.96)	<0.001
Procalcitonin, ng/mL	17.82 (63.71)	16.63 (72.22)	18.14 (54.56)	18.68 (69.98)	20.16 (60.38)	16.70 (36.43)	0.863
Creatinine, umol/L	147.93 (141.06)	143.50 (145.85)	167.66 (138.47)	141.56 (146.96)	155.67 (126.43)	151.10 (130.08)	0.018
Alanine Aminotransferase, U/L	151.78 (495.86)	136.81 (594.76)	141.95 (426.11)	166.87 (466.25)	181.73 (482.92)	137.91 (386.29)	0.407
Aspartate Aminotransferase, U/L	342.03 (1366.61)	294.81 (1344.24)	304.52 (1088.24)	372.06 (1515.66)	429.51 (1375.92)	338.62 (1251.62)	0.459
Total bilirubin, umol/L	63.79 (109.59)	69.26 (125.54)	42.45 (69.56)	67.21 (108.65)	62.43 (104.41)	61.03 (101.44)	0.001
White blood cell count, ×10^12^/L	13.16 (9.27)	12.86 (11.65)	14.17 (8.25)	12.89 (7.80)	13.51 (7.87)	13.39 (8.22)	0.123
Hemoglobin, g/L	86.22 (21.25)	84.33 (20.71)	89.55 (23.08)	87.29 (21.68)	85.04 (20.93)	86.46 (20.14)	<0.001
Platelet count, ×10^9^/L	143.93 (112.86)	147.05 (114.65)	146.73 (99.58)	139.25 (102.94)	139.14 (106.14)	148.41 (136.35)	0.323
Lactic acid, mmol/L	3.53 (3.35)	3.05 (2.92)	3.68 (2.70)	3.53 (3.63)	4.62 (3.83)	3.64 (3.39)	<0.001
Vital signs, mean (SD)							
Heart rate,/min	116.12 (22.31)	114.48 (20.60)	119.91 (21.70)	114.75 (22.73)	120.28 (23.73)	116.78 (23.32)	<0.001
Respiratory rate,/min	26.54 (7.64)	26.91 (7.51)	26.58 (8.38)	26.47 (7.30)	26.85 (8.26)	25.78 (7.61)	0.052
Mean arterial pressure, mmHg	62.54 (11.48)	63.62 (11.22)	61.87 (11.89)	62.52 (11.29)	61.54 (11.20)	61.69 (12.07)	0.002
Temperature, °C	37.40 (1.19)	37.36 (1.20)	37.63 (1.22)	37.37 (1.14)	37.28 (1.20)	37.48 (1.22)	<0.001
Central venous pressure, cmH_2_O	13.12 (7.53)	12.23 (6.92)	13.77 (8.02)	13.45 (7.89)	13.59 (7.32)	13.37 (7.58)	<0.001
Urine output, mL	1882.49 (1347.15)	1861.37 (1333.26)	1886.93 (1282.78)	1907.29 (1410.19)	1878.31 (1279.32)	1874.26 (1333.15)	0.953
Steroid therapy, %	475 (13.00)	113 (10.09)	54 (14.79)	149 (12.88)	55 (13.99)	104 (16.77)	0.002
Insulin dose, IU	171.66 (326.69)	61.69 (148.99)	506.20 (557.99)	107.32 (217.84)	282.11 (436.77)	223.42 (302.20)	<0.001
Organ support, %							
CRRT	1308 (35.79)	339 (30.27)	156 (42.74)	408 (35.26)	163 (41.48)	242 (39.03)	<0.001
MV	2769 (75.76)	769 (68.66)	292 (80.00)	909 (78.57)	312 (79.39)	487 (78.55)	<0.001
1-year mortality, n (%)	1674 (45.80)	438 (39.11)	204 (55.89)	503 (43.47)	203 (51.65)	326 (52.58)	<0.001

Cluster 0 (“low-stable”), cluster 1 (“high-stable”), cluster 2 (“moderate-stable”), cluster 3 (“high-decreasing”), cluster 4 (“moderate-increasing”).

SD, Standard Deviation; BMI, Body Mass Index; CCI, Charlson Comorbidity Index; ICU, intensive care unit; APACHE, Acute Physiologic Assessment and Chronic Health Evaluation; SOFA, Sequential Organ Failure Assessment; BG, blood glucose; CV, Coefficient of Variation; CRP, C Reactive Protein; CRRT, continuous renal replacement therapy; MV, Mechanical Ventilation; ×, multiplication.

### Blood glucose trajectory clustering

To determine the optimal number of clusters (K), multiple metrics were employed, as shown in **Supplementary Table S1** and **Supplementary Figure S2**. The elbow plot and SSE values suggested an “elbow” around K=5, where the incremental decrease of SSE became relatively small after this point (**Supplementary Figure S2**). Additionally, the Calinski-Harabasz index peaked at K=5, while the Silhouette Score remained satisfactory. Consequently, we selected K=5 as the optimal cluster number, as these distinct trends exhibited high internal similarity, with additional clusters offering limited gain in differentiation.

**Figure 2:**
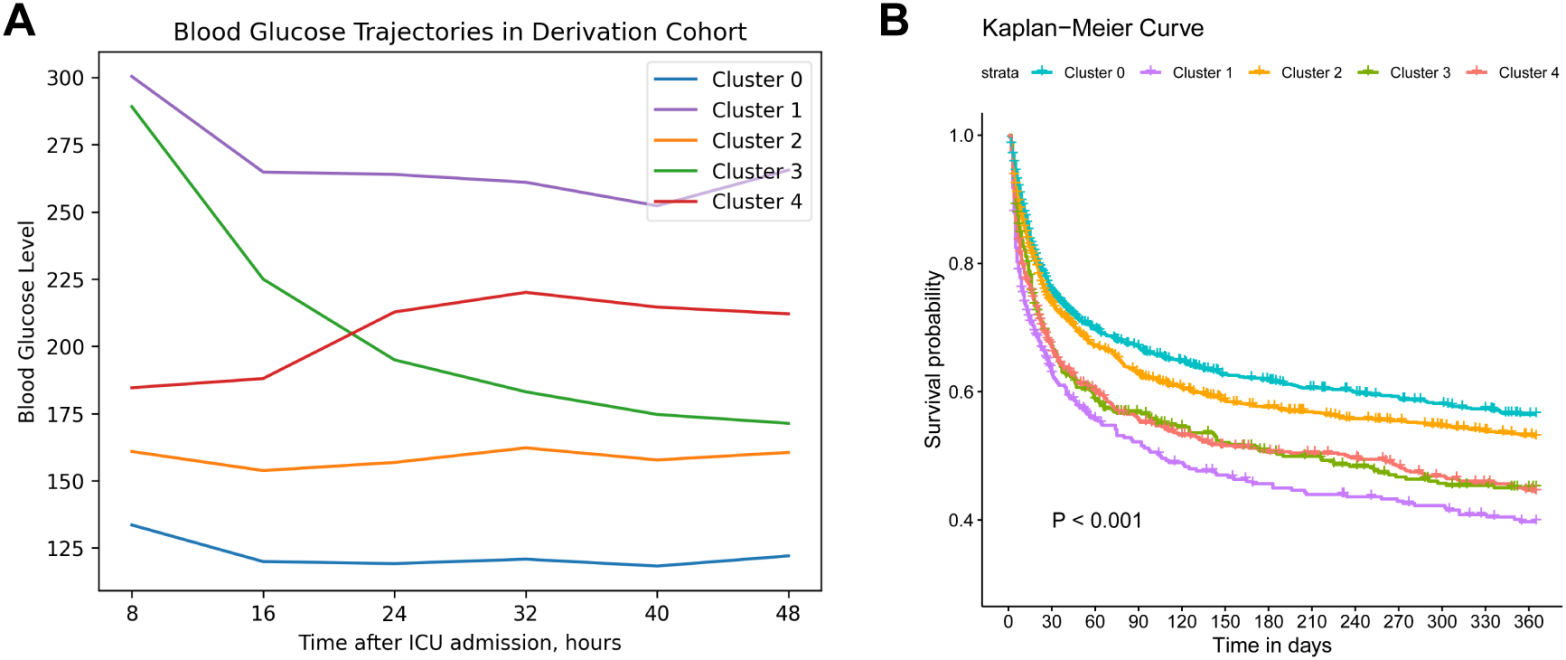
Trajectories and survival analysis of derivation cohort. **(A)**. Trajectories of blood glucose levels within 48 hours after ICU admission among patients diagnosed with sepsis in the derivation cohort (N=3,655). **(B)**. Kaplan–Meier survival curve of 1-year mortality across different blood glucose trajectory groups. Cluster 0 (“low-stable”), cluster 1 (“high-stable”), cluster 2 (“moderate-stable”), cluster 3 (“high-decreasing”), cluster 4 (“moderate-increasing”).

**Figure 2A** illustrates the longitudinal BG trajectories of the five identified clusters over the initial 48 hours following ICU admission. Cluster 0 (30.64%) displayed a relatively stable and low glucose trajectory over the entire course (“low-stable”), with a median value of 127.8 mg/dL (IQR: 108.0–149.0) at baseline and 120.6 mg/dL (IQR: 104.4–136.8) at 48 hours. Cluster 1 (9.99%) maintained high glucose levels throughout the period (“high-stable”), with a median decrease from 288.0 mg/dL (IQR: 241.2–347.4) to 255.6 mg/dL (IQR: 223.2–302.4), corresponding to an absolute reduction of about 32.4 mg/dL. Cluster 2 (31.66%) started at a moderate glucose level and remained stable (“moderate-stable”), showing only a slight decline from 158.4 mg/dL (IQR: 135.0–183.6) to 156.6 mg/dL (IQR: 138.6–178.2). In contrast, Cluster 3 (10.75%) presented with a high initial glucose level but showed a pronounced drop over the 48-hour period (“high-decreasing”), with values falling from 271.8 mg/dL (IQR: 239.4–320.4) to 167.4 mg/dL (IQR: 140.4–198.0), representing an absolute decrease of approximately 104.4 mg/dL and about 39% reduction from baseline. Lastly, cluster 4 (16.96%) was characterized by starting at a moderate level and then increasing (“moderate-increasing”), which showed an upward trend in BG, increasing from a median of 183.6 mg/dL (IQR: 153.0–214.2) to 205.2 mg/dL (IQR: 180.0–237.6), with an overall increase of 21.6 mg/dL.

### Baseline characteristics and outcomes of different clusters

The baseline characteristics of these clusters are summarized in **Table 1**, revealing diverse demographics, vital signs, blood counts and biochemistry profiles. Notably, cluster 1 (“high-stable”) had the highest mean glucose levels over the period (268.06 mg/dL), whereas glycemic variability in cluster 3 (“high-decreasing”) was the greatest (CV: 0.28), further supporting the clinical relevance of our clustering approach. Additionally, cluster 1 exhibited the highest mean APACHE-II score (23.21) and 1-year mortality (55.89%), followed by cluster 4 (“moderate-increasing”) and cluster 3 (52.58%, 51.65%, respectively). Cluster 0 (“low-stable”) was related to the lowest 1-year mortality (39.11%). Kaplan-Meier curves of all clusters are illustrated in **Figure 2B**, indicating that cluster 1 showed the worst 1-year survival (log-rank *P* < 0.001).

Primary and secondary outcomes across these clusters are displayed in **Table 2**. There was no significant difference between these trajectories and ICU length of stay (*P* = 0.165). The trends of ICU and 30-day mortality across the subtypes were similar to 1-year results. Specifically, individuals in cluster 3 were associated with the largest amount of vasopressor usage (50 mg, IQR [10, 148]), along with the longest ventilation durations (106 hours, IQR [51, 212]), suggesting a greater need for organ support and hemodynamic stabilization for those with a “high-decreasing” glucose trajectory. Additionally, cluster 1 (“high-stable”) received the highest doses of insulin (320 IU, IQR [168, 580]).

**Table 2:** Primary and secondary outcomes across different blood glucose trajectory clusters.

Variables	Cluster 0	Cluster 1	Cluster 2	Cluster 3	Cluster 4	*P* Value
Primary outcome						
1-year mortality, %	438 (39.18)	204 (55.89)	503 (43.47)	203 (51.65)	326 (52.67)	<0.001
Secondary outcome						
ICU length of stay^*^, hours	141 (83, 271)	143 (93, 296)	140 (88, 253)	159 (95, 318)	137 (90, 257)	0.165
ICU mortality, %	167 (14.94)	104 (28.49)	202 (17.46)	94 (23.92)	154 (24.88)	<0.001
30-day mortality, %	265 (23.70)	133 (36.44)	295 (25.50)	127 (32.32)	204 (32.96)	<0.001
Vasopressors^*^, mg	20 (0, 90)	40 (10, 142)	30 (0, 110)	50 (10, 148)	40 (9.50, 130)	<0.001
CRRT, %	339 (30.27)	156 (42.74)	408 (35.26)	163 (41.48)	242 (39.03)	<0.001
Ventilation duration^*^, hours	72 (21, 173)	102 (61, 187)	76 (34, 173)	106 (51, 212)	91 (41, 186)	<0.001
Insulin^*^, IU	6 (0, 58)	320 (168, 580)	25 (0, 104)	144 (40, 360)	120 (40, 280)	<0.001

^*^Median (IQR); ICU, intensive care unit; CRRT, continuous renal replacement therapy.

Table legend: Cluster 0 (“low-stable”), cluster 1 (“high-stable”), cluster 2 (“moderate-stable”), cluster 3 (“high-decreasing”), cluster 4 (“moderate-increasing”).

### Multivariate Cox regression models

Univariate analysis revealed significant risk factors associated with 1-year mortality in sepsis patients, including age, gender, admission department, CCI, APACHE II, SOFA, creatinine, total bilirubin, hemoglobin, heart rate, mean arterial pressure, and urine output. The association between blood glucose trajectories and 1-year mortality risks in different Cox proportional hazards regression models is presented in **Table 3**. Both unadjusted and adjusted hazard ratios demonstrated that cluster 1 (“high-stable”) was related to a significantly higher risk of 1-year mortality compared to cluster 0 (“low-stable”) (HR = 1.68, 1.63, 1.61 in the univariate model, model 1, and model 2, respectively; all *P* < 0.001). Similarly, both cluster 3 (“high-decreasing”) and cluster 4 (“moderate-increasing”) were linked to increased 1-year mortality risks in the full multivariate model (HR: 1.38, 95% CI: 1.16-1,65, *P* < 0.001; HR: 1.37, 95% CI: 1.18-1.60, *P* < 0.001, respectively). No significant difference was found in the 1-year mortality risk of cluster 2 (“moderate-stable”) compared to the reference group (*P* = 0.077 in model 2).

**Table 3:** Univariate and multivariate Cox regression models of 1-year mortality risk.

Variables	HR	95%CI		*P* Value
Univariate model				
Cluster 0	Ref			
Cluster 1	1.68	1.42	1.98	<0.001
Cluster 2	1.12	0.98	1.27	0.086
Cluster 3	1.42	1.2	1.68	<0.001
Cluster 4	1.45	1.25	1.67	<0.001
Model 1				
Cluster 0	Ref			
Cluster 1	1.63	1.37	1.92	<0.001
Cluster 2	1.10	0.96	1.25	0.162
Cluster 3	1.40	1.19	1.66	<0.001
Cluster 4	1.41	1.22	1.63	<0.001
Model 2				
Cluster 0	Ref			
Cluster 1	1.61	1.35	1.92	<0.001
Cluster 2	1.13	0.99	1.29	0.077
Cluster 3	1.38	1.16	1.65	<0.001
Cluster 4	1.37	1.18	1.60	<0.001

Cluster 0 (“low-stable”), cluster 1 (“high-stable”), cluster 2 (“moderate-stable”), cluster 3 (“high-decreasing”), cluster 4 (“moderate-increasing”).

Model 1: adjusted for age, gender.

Model 2: adjusted for age, gender, admission department, CCI, APACHE II, SOFA, creatinine, total bilirubin, hemoglobin, heart rate, mean arterial pressure, urine output.

### External validation

**Figure 3:**
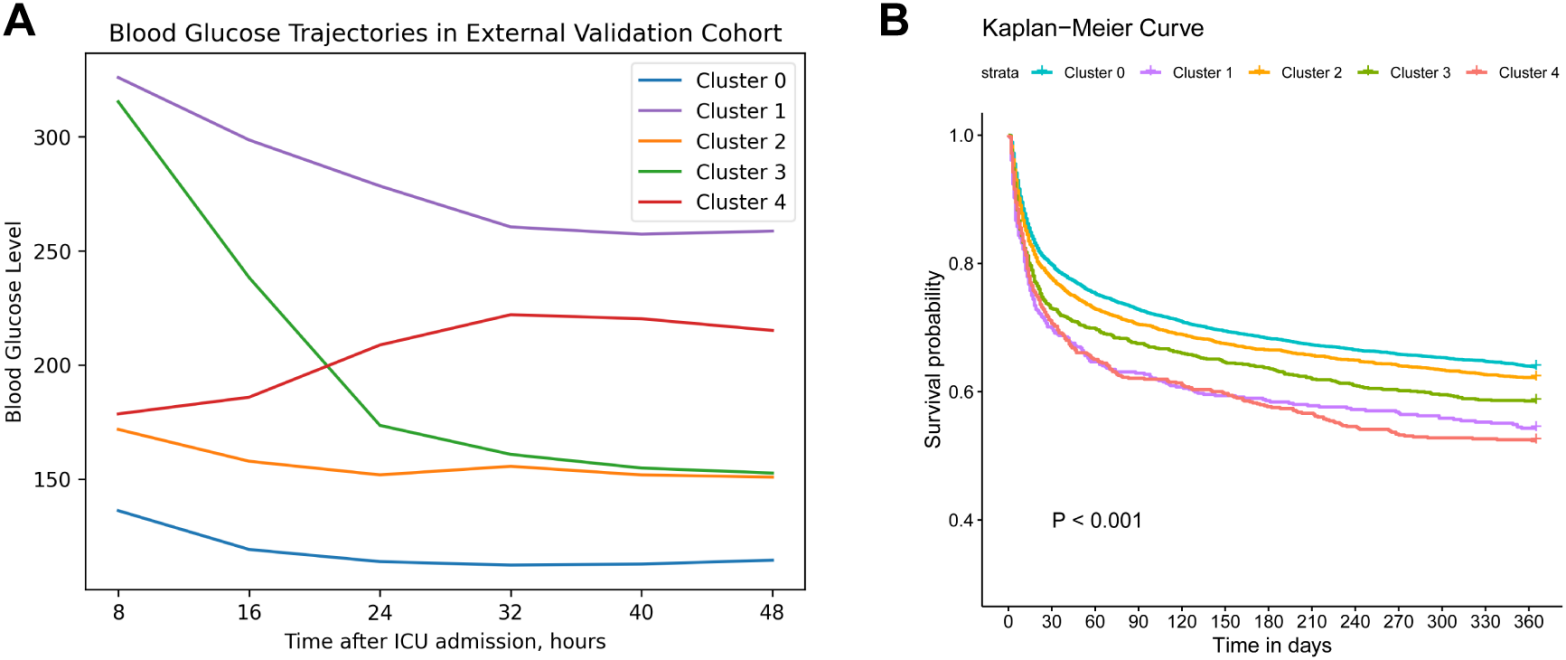
External validation clustering results. **(A)**. Trajectory validation of blood glucose levels within 48h after ICU admission in MIMIC IV database. **(B)**. Kaplan–Meier survival curve of 1-year mortality across different trajectories. Cluster 0 (“low-stable”), cluster 1 (“high-stable”), cluster 2 (“moderate-stable”), cluster 3 (“high-decreasing”), cluster 4 (“moderate-increasing”).

To externally validate our clustering approach, we applied the same process to 10,874 sepsis patients from the MIMIC-IV database, with the selection process outlined in **Supplementary Figure S1B**. Baseline characteristics are summarized in **Supplementary Table S2**. Consistent clustering subtypes were obtained in the validation cohort: cluster 0 (“low-stable”, 53.27%), cluster 1 (“high-stable”, 4.71%), cluster 2 (“moderate-stable”, 25.51%), cluster 3 (“high-decreasing”, 10.27%), cluster 4 (“moderate-increasing”, 6.24%). As displayed in **Figure 3**, cluster 0 exhibited the most favorable 1-year survival, whereas both cluster 1 and cluster 4 showed the worst 1-year survival outcomes (log-rank P<0.001).

**Figure 4:**
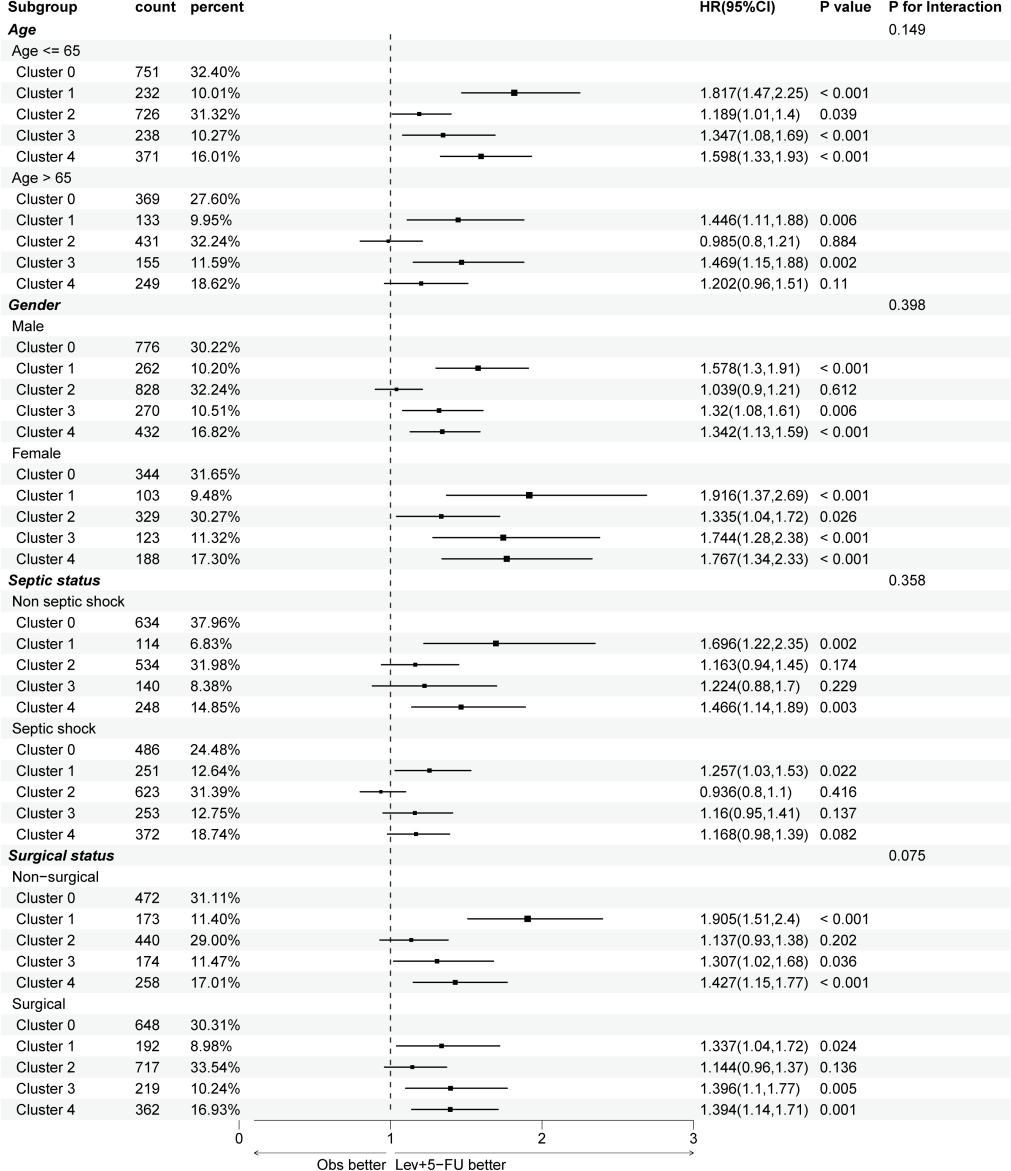
Subgroup analysis of 1-year mortality risks across different trajectories, stratifying by age, gender, septic shock, and surgical status. APACHE, acute physiologic assessment and chronic health evaluation; HR, hazard ratio. Cluster 0 (“low-stable”), cluster 1 (“high-stable”), cluster 2 (“moderate-stable”), cluster 3 (“high-decreasing”), cluster 4 (“moderate-increasing”).

### Subgroup and sensitivity analyses

To control baseline imbalances across the formed clusters, subgroup analyses were conducted in the derivation cohort, as illustrated in **Figure 4**. The association between blood glucose trajectories and 1-year mortality risk remained largely consistent regardless of gender and surgical status, where a “low-stable” or “moderate-stable” glucose trend was correlated with better outcomes. In aging patients (>65 years), only cluster 1 (“high-stable”) and cluster 3 (“high-decreasing”) showed significant long-term mortality risks (P=0.006, 0.002, respectively), suggesting that high glucose levels at admission may indicate poor outcomes for elderly individuals. Additionally, in septic shock patients, only cluster 1 exhibited a significantly worse prognosis (P=0.022), which could be related to increased oxidative stress, coagulation activation, and endothelial dysfunction as a result of persistent stress hyperglycemia.

To validate the impact of diabetes history on the BG clustering process, sensitivity analysis was performed in diabetes and non-diabetes patients, respectively. In patients without diabetes history (N=2,981), the survival outcomes across trajectories were similar to the main results (**Supplementary Figures S3A, B**). However, in diabetic patients, the blood glucose trajectories were similar to the primary analysis, whereas 5 clusters showed different 1-year mortality. Cluster 0 (“low-stable”) presented the worst 1-year prognosis, though there was no statistically significant difference in 1-year mortality rate among the five groups (log-rank *P* = 0.2) (**Supplementary Figure S3C, D**). This suggests that diabetic patients may have a distinct immune response and metabolism as a consequence of the host’s chronic tolerance to hyperglycemia.

**Figure 5:**
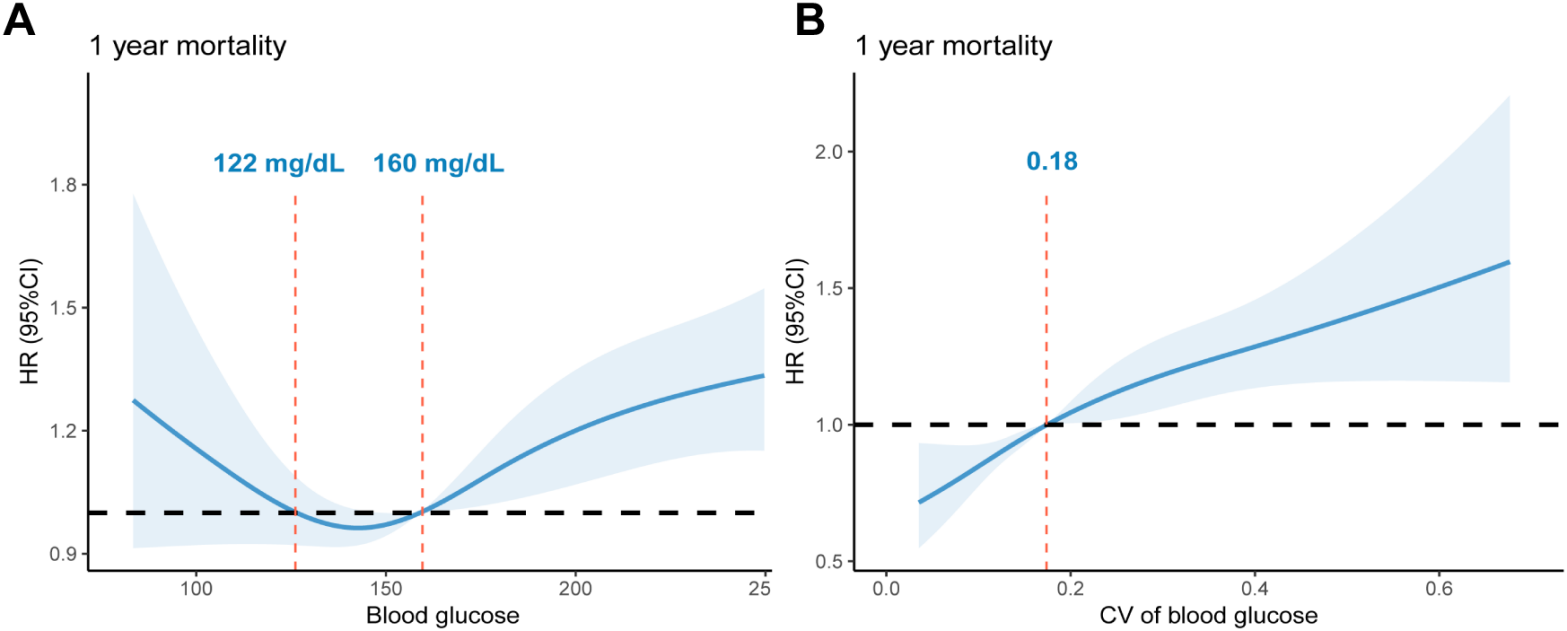
RCS curves of glycemic indexes associated with 1-year mortality risk: **(A)**. RCS curves of mean blood glucose within 48 hours after ICU admission. The reference glucose levels are between 122 mg/dL and 160 mg/dL. **(B)**. RCS curves of glycemic CV within 48 hours after ICU admission. The reference value of CV: 0.18. RCS, restricted cubic spline; CV, Coefficient of Variation; HR, hazard ratio.

Lastly, RCS was used to further explore the optimal mean blood glucose and CV ranges associated with lower 1-year mortality risks (**Figure 5**). Both blood glucose and CV exhibited a nonlinear relationship with 1-year mortality (*P* for nonlinear < 0.001, both). There was a U-shaped curve between the mean blood glucose values within 48 hours and 1-year mortality, with an optimal range between 122 mg/dL and 160 mg/dL. Contrarily, the relationship between CV and 1-year mortality exhibited a J-shaped curve, with a higher risk of death when CV was greater than 0.18.

## Discussion

We identified and externally validated five distinct early blood glucose trajectories in sepsis patients, namely, the “low-stable”, “high-stable”, “moderate-stable”, “high-decreasing”, and “moderate-increasing” trends. Additionally, these BG trajectories were significantly associated with diverse 1-year mortality risks, supporting the notion that early dynamic glucose patterns may play a crucial role in long-term sepsis prognosis. The five trajectories were also reproducible in the MIMIC-IV database.

Sepsis is a heterogeneous disease with high morbidity and mortality that rapidly progresses, thus identifying prognostic markers in the early stage could assist in risk stratification and individualized management. Given the complex interplay between sepsis progression and glucose metabolism, numerous investigations have focused on the relationship between blood glucose levels and sepsis outcomes (17, 27–30). However, most previous studies were based on cross-sectional data, typically assessing the glycemic indexes at a specific time point. For instance, Lu et al. utilized the mean glucose values and glycemic CV during the ICU stay to examine their impacts on ICU mortality among 7,104 adult sepsis patients (30). The multivariate logistic regression results showed that both increased glucose levels and higher CV were correlated with increased mortality risks (OR: 1.14, 1.05, respectively), while the harm of hyperglycemia was not observed in diabetic patients. However, this approach neglects the dynamic nature of glucose metabolism and its potential effect on sepsis outcomes. Our study used time-series data of varying blood glucose trajectories and applied K-means clustering to deconstruct the heterogeneity, providing a more granular view of early glycemic patterns, in contrast to single snapshot measurements. We identified five distinct clusters of early BG trajectories, linking to different 1-year outcomes. The most favorable prognosis was observed in patients with “low-stable” and “moderate-stable” trajectories, whereas individuals with a “high-stable” trajectory and those exhibiting unstable trends (“high-decreasing” and “moderate-increasing”) showed significantly higher 1-year mortality risks. These findings were consistent with survival analysis and multivariate Cox proportional hazards regression results.

Several potential mechanisms may explain these findings. The “high-stable” trajectory may reflect persistent stress hyperglycemia and insulin resistance, which has been found to be associated with increased oxidative stress, coagulation activation, and endothelial dysfunction (31). Similarly, the dynamic increase of the triglyceride-glucose (TyG) index trajectory, an indicator of insulin resistance, is also reported to be associated with higher 28-day mortality risk (HR: 1.07, 95% CI: 1.02-1.12) (32). Moreover, greater glycemic variability, as observed in two clusters with unstable trends, has been linked to adverse outcomes in critically ill patients. Previous research has suggested that apoptosis in endothelial cells was even more severe with an acute fluctuating glucose exposure than with a stable high glucose concentration (33, 34). Besides, GV could also increase the occurrence of severe hypoglycemia, leading to mortality, prolonged hospital length of stay, and an increased risk of cardiovascular events for sepsis patients (35, 36). Importantly, our RCS analysis further supports these observations, which revealed a U-shaped association for mean glucose values, and a J-shaped relationship for GV linked to 1-year mortality, thus indicating a detrimental effect of extreme glycemic levels and excessive glucose fluctuations.

To ensure the consistency of our findings, we performed external validation in a demographically diverse cohort, demonstrating the reproducibility of our clustering technique. Despite the similarities in trajectory clustering, slight differences in 1-year mortalities were noticed between the FAH-SYSU and MIMIC cohorts. For instance, cluster 4 (“moderate-increasing”) exhibited even worse 1-year survival than cluster 1 (“high-stable”) in the MIMIC cohort. Variations in mortalities across clusters suggest that latent factors, such as patient demographics, insulin protocols, vasopressor usage, and the implementation of advanced organ support, may influence outcomes. Moreover, we conducted subgroup and sensitivity analyses, revealing that the relationship between BG trajectories and 1-year mortality remained largely consistent across different age groups, genders, and sepsis subtypes. Notably, survival analysis demonstrated that in diabetic patients, a “low-stable” trajectory might indicate the worst 1-year prognosis in contrast to non-diabetic patients. Lin et al. found that severe hyperglycemia did not increase the risk of 28-day mortality among 3,500 sepsis patients with diabetes (HR: 1.06, 95% CI: 0.86-1.31), while a lower admission blood glucose level was associated with increased risk of poor prognosis (29). Physiologically, patients with diabetes have chronically grown tolerance to hyperglycemia with impaired β-cell function, thus a “low-stable” trajectory may signal treatment-induced hypoglycemia. It is argued that various rapidly dividing cells, including immune cells, rely on aerobic glycolysis as a normal metabolic strategy (37). For patients with diabetes, elevated glucose levels may play a vital role in maintaining biosynthetic activities associated with the expansion of immune cells and the production of immune modulators during critical conditions (29). Moreover, insulin administration may adversely affect clinical outcomes by inhibiting autophagy, which could lead to the inability to remove damaged proteins or degrade noxious factors including bacteria and endotoxins (37). Our findings further highlight the importance of continuous blood glucose monitoring and individualized glycemic control strategies based on the patient’s comorbidities. Future studies should explore whether interventions targeting specific BG trajectories can improve survival in various sepsis patients.

Our study has several strengths. First, it is the largest investigation to our knowledge examining the relationship between hyperglycemia BG trajectories and long-term outcomes in sepsis patients, while previous investigations have largely focused on snapshot glycemic measurements and prognoses in the short-to-medium term. Second, the use of unsupervised machine learning (K-means clustering) and the consensus approach utilizing both quantitative metrics and qualitative inspection to determine the optimal K provide an objective classification of BG patterns without predefined assumptions, thereby ensuring that the formation of clusters can best explain the data. Third, the validation in a demographically diverse and independent cohort further strengthens the generalizability of our findings.

However, the limitations should also be acknowledged. First, this study is retrospective in nature; therefore, biases in data collection and patient selection may have occurred during the database construction and information extraction, potentially affecting the accuracy and reliability of our clusters. Nevertheless, we have utilized internal and external validation to ensure the credibility of the clustering approach, and conducted various statistical analyses to account for potential confounders. Second, due to the requirement of calculating dynamic trajectory, patients who stayed less than 48 hours were excluded, thus individuals with milder conditions or those who died before the endpoint were not considered in our clustering process. The exclusion of these patients may lead to neglect in identifying certain BG patterns. Additionally, we acknowledge the inherent limitations of the K-means clustering algorithm. K-means assumes spherical clusters of equal variance and is sensitive to initial centroid placement. It also requires pre-specifying the number of clusters (k), which may not perfectly capture the true underlying data structure. While we addressed this by using multiple metrics including SSE, Silhouette Score, Calinski-Harabasz index, and Davies-Bouldin index to determine an optimal k, more advanced trajectory modeling techniques such as group-based trajectory modeling or latent class mixed models may offer improved flexibility in future work. In our study, 13% of patients in the derivation cohort received steroid therapy within the first 48 hours after ICU admission, which may contribute to hyperglycemia or glycemic variabilityFuture prospective studies should incorporate detailed treatment data, including insulin protocols, corticosteroid dose, and nutritional interventions, to better understand how clinical interventions shape blood glucose trajectories and long-term outcomes.

## Conclusion

Distinct trajectories of early blood glucose dynamic change were significantly associated with 1-year mortality in patients with sepsis, while individuals with persistent hyperglycemia and unstable glucose trends showed significantly higher 1-year mortality risks after adjusting for confounders. By focusing on the early post-admission period, our findings highlight the prognostic value of dynamic BG measurements, offering a novel approach to predict clinical outcomes in sepsis.

## Data Availability

The datasets used and/or analyzed during the current study are available from the corresponding authors on reasonable request.

## Supplementary material

Supplementary Figure 1 Flow diagram of patient selection in the derivation cohort **(A)** and validation cohort **(B)**.

Supplementary Figure 2 The elbow plot of K-means clustering results.

Supplementary Figure 3 Sensitivity analysis among patients with and without diabetes. **(A)**. Trajectory of blood glucose levels within 48h after ICU admission in non-diabetic patients. **(B)**. 1-year Kaplan–Meier survival curve of non-diabetic patients. **(C)**. Trajectory of blood glucose levels within 48h after ICU admission in diabetic patients. **(D)**. 1-year Kaplan–Meier survival curves of diabetic patients. Cluster 0 (“low-stable”), cluster 1 (“high-stable”), cluster 2 (“moderate-stable”), cluster 3 (“high-decreasing”), cluster 4 (“moderate-increasing”).

Supplementary Table 1 Performance metrics of K-means clustering with different number of clusters (K).

Supplementary Table 2 Demographic and clinical characteristics of external validation cohort from the MIMIC-IV database. Cluster 0 (“low-stable”), cluster 1 (“high-stable”), cluster 2 (“moderate-stable”), cluster 3 (“high-decreasing”), cluster 4 (“moderate-increasing”).

Appendix 1 CAIR Checklist for Healthcare AI Research.
