# Supplementary material for: Identifying early blood glucose trajectories in sepsis linked to distinct long-term outcomes: a K-means clustering study with external validation
Source: Front Immunol. 2025 Jun 5;16:1610519. doi: 10.3389/fimmu.2025.1610519 (PMC12176745; doi:10.3389/fimmu.2025.1610519)
Supplement: Supplementary Figure 1 — Flow diagram of patient selection in the derivation cohort (A) and validation cohort (B). [file SupplementaryFile1.docx]

**Supplementary information**

**Figure S1.** Flow diagram of patient selection in the derivation cohort (A) and validation cohort (B).


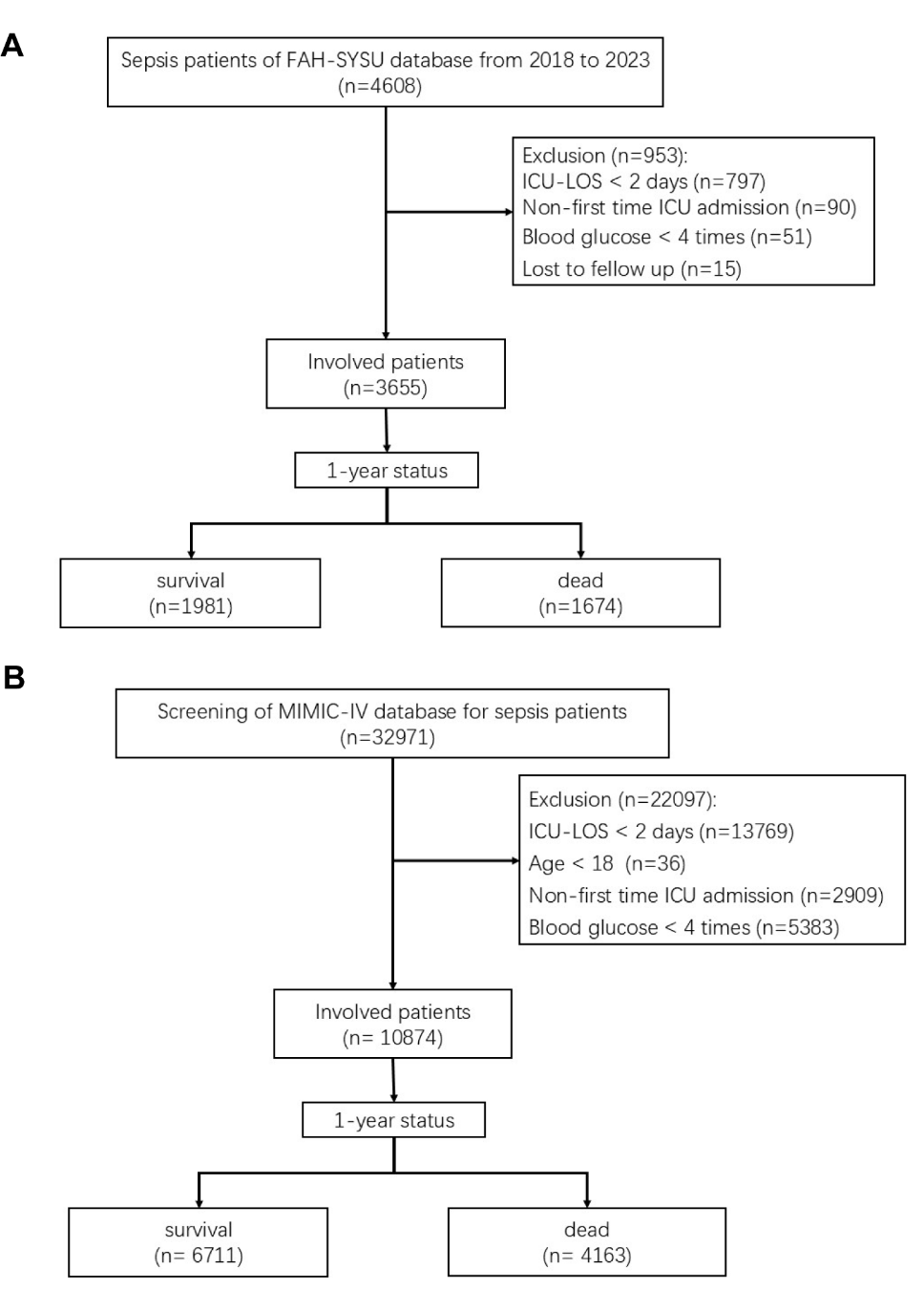


**Figure S2.** The elbow plot of K-means clustering results.


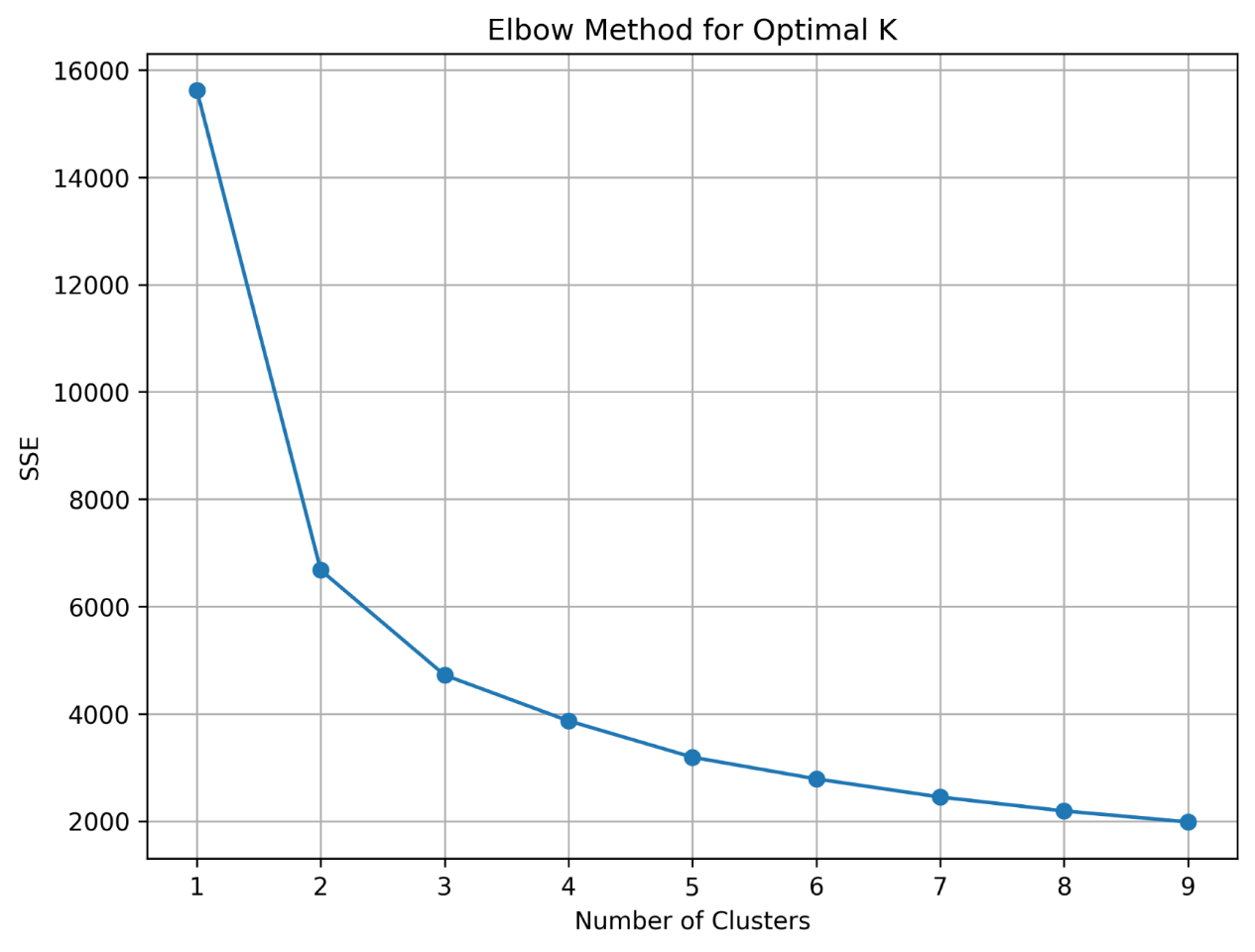


**Figure S3.** Sensitivity analysis among patients with and without diabetes. (A). Trajectory of blood glucose levels within 48h after ICU admission in non-diabetic patients. (B). 1-year Kaplan–Meier survival curve of non-diabetic patients. (C). Trajectory of blood glucose levels within 48h after ICU admission in diabetic patients. (D). 1-year Kaplan–Meier survival curves of diabetic patients. Cluster 0 (“low-stable”), cluster 1 (“high-stable”), cluster 2 (“moderate-stable”), cluster 3 (“high-decreasing”), cluster 4 (“moderate-increasing”).

**
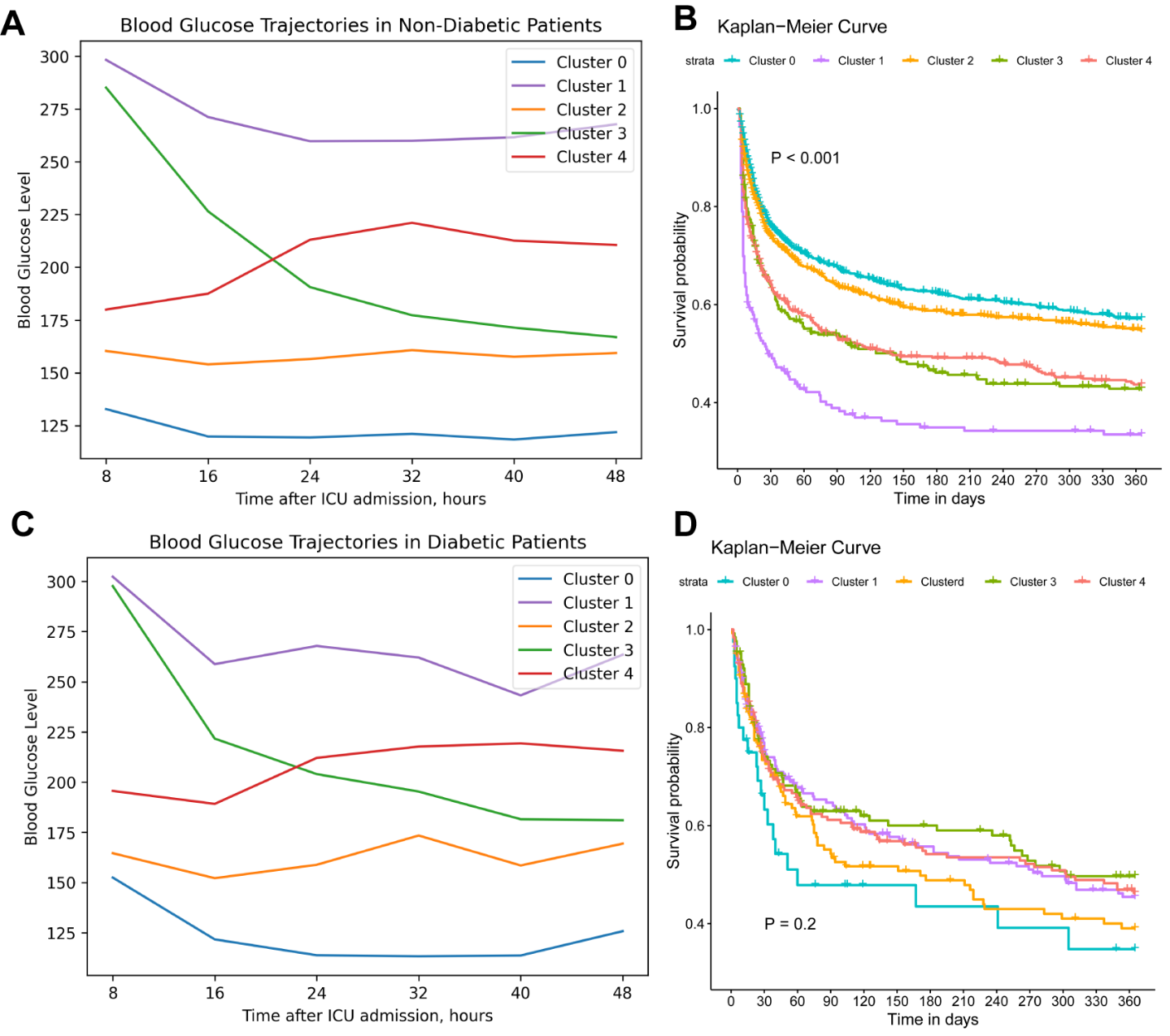
Table S1.** Performance metrics of K-means clustering with different number of clusters (K).

| **K** | **SSE** | **Silhouette Score** | **Calinski-Harabasz** | **Davies-Bouldin** | **Cluster 0, %** | **Cluster 1, %** | **Cluster 2, %** | **Cluster 3, %** | **Cluster 4, %** | **Cluster 5, %** |
| --- | --- | --- | --- | --- | --- | --- | --- | --- | --- | --- |
| **1** | 15618 |  |  |  | 100 |  |  |  |  |  |
| **2** | 6676 | 0.338 | 3560.271 | 0.748 | 34.64 | 65.36 |  |  |  |  |
| **3** | 4720 | 0.349 | 3702.782 | 0.961 | 35.87 | 47.49 | 16.64 |  |  |  |
| **4** | 3867 | 0.332 | 4221.969 | 1.002 | 33.42 | 23.83 | 12.27 | 30.49 |  |  |
| **5** | 3190 | 0.409 | 4899.387 | 0.933 | 30.64 | 9.99 | 31.66 | 10.75 | 16.96 |  |
| **6** | 2885 | 0.512 | 4513.106 | 0.891 | 27.98 | 14.78 | 15.46 | 10.11 | 25 | 6.67 |

SSE, Sum of Squared Errors;

**Table S2.** Demographic and clinical characteristics of external validation cohort from the MIMIC-IV database. Cluster 0 (“low-stable”), cluster 1 (“high-stable”), cluster 2 (“moderate-stable”), cluster 3 (“high-decreasing”), cluster 4 (“moderate-increasing”).

| **Variables** | **Overall**  **n = 10874** | **Cluster 0**  **n = 5793** | **Cluster 1**  **n = 512** | **Cluster 2**  **n = 2774** | **Cluster 3**  **n = 1117** | **Cluster 4**  **n = 678** | ***P* Value** |
| --- | --- | --- | --- | --- | --- | --- | --- |
| **Age, mean (SD), y** | 65.35 (15.95) | 64.75 (16.99) | 64.74 (14.01) | 66.82 (14.95) | 64.41 (14.58) | 66.51 (13.76) | <0.001 |
| **Sex, n (%)** |  |  |  |  |  |  | 0.441 |
| **Female** | 4511 (41.48) | 2434 (42.02) | 202 (39.45) | 1124 (40.52) | 457 (40.91) | 294 (43.36) |  |
| **Male** | 6363 (58.52) | 3359 (57.98) | 310 (60.55) | 1650 (59.48) | 660 (59.09) | 384 (56.64) |  |
| **CCI, mean (SD)** | 5.36 (2.93) | 5.01 (2.93) | 6.47 (2.81) | 5.48 (2.93) | 5.70 (2.68) | 6.43 (2.81) | <0.001 |
| **Race, n (%)** |  |  |  |  |  |  | <0.001 |
| **ASIAN** | 297 (2.73) | 146 (2.52) | 14 (2.73) | 77 (2.78) | 40 (3.58) | 20 (2.95) |  |
| **BLACK** | 1052 (9.67) | 523 (9.03) | 63 (12.30) | 245 (8.83) | 149 (13.34) | 72 (10.62) |  |
| **WHITE** | 7681 (70.64) | 4168 (71.95) | 347 (67.77) | 1958 (70.58) | 725 (64.91) | 483 (71.24) |  |
| **OTHER** | 1844 (16.96) | 956 (16.50) | 88 (17.19) | 494 (17.81) | 203 (18.17) | 103 (15.19) |  |
| **Severity score, mean (SD)** |  |  |  |  |  |  |  |
| **SOFA** | 4.10 (2.26) | 4.01 (2.21) | 4.04 (2.19) | 4.09 (2.17) | 4.52 (2.55) | 4.21 (2.40) | <0.001 |
| **SAPSII** | 44.20 (14.32) | 42.72 (13.73) | 47.72 (15.43) | 44.40 (14.23) | 48.32 (15.23) | 46.51 (15.14) | <0.001 |
| **Laboratory result, mean (SD)** |  |  |  |  |  |  |  |
| **BG on admission, mg/dL** | 155.35 (93.03) | 121.56 (44.32) | 310.48 (176.31) | 150.72 (68.29) | 261.12 (129.45) | 170.19 (69.29) | <0.001 |
| **Mean BG in 48hours, mg/dL** | 150.38 (46.50) | 118.70 (16.50) | 281.61 (35.78) | 157.30 (11.52) | 204.89 (24.88) | 203.85 (16.99) | <0.001 |
| **CV of BG in 48hours** | 0.22 (0.13) | 0.18 (0.11) | 0.29 (0.14) | 0.20 (0.11) | 0.38 (0.15) | 0.24 (0.15) | <0.001 |
| **Prothrombin time, s** | 19.78 (14.15) | 19.57 (13.54) | 19.86 (14.35) | 19.40 (13.52) | 20.97 (17.47) | 21.10 (15.36) | 0.002 |
| **Creatinine, mg/dL** | 2.03 (2.07) | 1.95 (1.99) | 2.58 (3.90) | 1.90 (1.73) | 2.27 (1.94) | 2.41 (2.03) | <0.001 |
| **Aspartate Aminotransferase, IU/L** | 454.05 (1763.79) | 384.34 (1525.28) | 462.43 (1461.56) | 449.86 (1878.47) | 773.51 (2447.74) | 413.90 (1741.42) | <0.001 |
| **Alanine Aminotransferase, IU/L** | 259.85 (1194.91) | 246.05 (1422.83) | 233.81 (659.94) | 234.66 (853.32) | 390.08 (1047.89) | 241.32 (912.17) | 0.015 |
| **Total bilirubin, mg/dL** | 2.42 (5.32) | 2.71 (5.76) | 1.41 (2.95) | 2.24 (5.18) | 1.79 (3.36) | 2.85 (6.38) | 0.001 |
| **White blood cell count, *10^12^/L** | 16.85 (13.75) | 15.86 (12.09) | 18.12 (11.06) | 17.89 (17.46) | 19.12 (13.81) | 16.37 (10.44) | <0.001 |
| **Hemoglobin, g/dL** | 9.45 (2.16) | 9.39 (2.10) | 10.07 (2.27) | 9.43 (2.14) | 9.51 (2.36) | 9.49 (2.26) | <0.001 |
| **Platelet count, *10^9^/L** | 170.38 (103.30) | 169.20 (104.36) | 184.54 (101.28) | 168.42 (99.66) | 168.59 (102.78) | 180.75 (109.88) | 0.001 |
| **Lactic acid, mmol/L** | 1.80 (1.41) | 1.66 (1.26) | 2.55 (2.11) | 1.79 (1.35) | 2.14 (1.62) | 2.00 (1.56) | <0.001 |
| **Vital signs, mean (SD)** |  |  |  |  |  |  |  |
| **Heart rate, /min** | 88.47 (16.80) | 87.86 (16.70) | 92.20 (17.16) | 88.06 (16.33) | 90.32 (17.29) | 89.43 (17.83) | <0.001 |
| **Respiratory rate, /min** | 19.97 (4.10) | 19.61 (4.09) | 21.48 (4.19) | 20.00 (3.90) | 20.64 (4.08) | 20.77 (4.40) | 0.052 |
| **Mean arterial pressure, mmHg** | 76.03 (9.95) | 75.66 (9.81) | 76.92 (10.59) | 76.15 (10.07) | 77.38 (10.04) | 75.71 (9.78) | 0.002 |
| **Temperature, ℃** | 36.87 (0.68) | 36.87 (0.63) | 36.92 (0.93) | 36.89 (0.66) | 36.78 (0.90) | 36.91 (0.63) | <0.001 |
| **Organ support, %** |  |  |  |  |  |  |  |
| **CRRT** | 1603 (14.74) | 763 (13.17) | 105 (20.51) | 373 (13.45) | 207 (18.53) | 155 (22.86) | <0.001 |
| **MV** | 7394 (68.00) | 3907 (67.44) | 281 (54.88) | 1985 (71.56) | 798 (71.44) | 423 (62.39) | <0.001 |
| **1-year mortality, n (%)** | 4163 (38.28) | 2094 (36.15) | 234 (45.70) | 1049 (37.82) | 463 (41.45) | 323 (47.64) | <0.001 |

SD, Standard Deviation; BMI, Body Mass Index; CCI, Charlson Comorbidity Index; ICU, intensive care unit; APACHE, Acute Physiologic Assessment and Chronic Health Evaluation; SOFA, Sequential Organ Failure Assessment; BG, blood glucose; CV, Coefficient of Variation; CRP, C Reactive Protein; CRRT, continuous renal replacement therapy; MV, Mechanical Ventilation;

# Appendix 1. CAIR Checklist for Healthcare AI Research

| Section | Item | Description | Filled for This Study |
| --- | --- | --- | --- |
| TITLE AND ABSTRACT | T1 | Explicitly mention 'machine learning' in the abstract to comply with AI disclosure recommendations. | The title specifies “K-means clustering”, a recognized machine learning method, and the abstract clearly states the use of machine learning in the context of sepsis prognosis. |
| \| INTRODUCTION \| \| --- \|  \|  \| \| --- \| | I1 | Describe the clinical problem and explain how the AI tool is used to address it in the clinical pathway. | The introduction discusses the challenge of glycemic dysregulation and uncertainty in optimal BG control in sepsis. The study positions machine learning (K-means clustering) as a tool to identify early glucose trajectory phenotypes to enhance long-term prognostication and inform individualized ICU glucose management. |
| METHODS | M1 | **State inclusion and exclusion criteria, at the participant and the input data level, separately.** State why these criteria were used. How were rare pathologies handled? | **Participant level:** Adult ICU patients (≥18 years) diagnosed with sepsis according to Sepsis-3 criteria (suspected infection and SOFA score increase ≥2) were included. Only the first ICU admission per patient was considered. Exclusion criteria included ICU stay <48 hours, <4 blood glucose measurements within the first 48 hours, unplanned discharge for non-medical reasons, and lack of follow-up data. These criteria ensured sufficient clinical observation time and completeness of outcome and input data for robust modeling.  **Input data level:** Inclusion required at least four blood glucose measurements within the first 48 hours to enable early trajectory analysis. Patients with insufficient measurement frequency or missing follow-up were excluded to avoid bias from incomplete data.  **Rare pathologies:** Rare pathologies were not explicitly included in the research cohort. Therefore, the study population does not involve such conditions. |
|  | M2 | **Describe how the input data was acquired, selected, and handled, and include any form of preprocessing before analysis.** If there were some specific considerations in handling the data, this should also be specified. | **Data acquisition:** Data were obtained from two independent cohorts: the FAH-SYSU ICU database (10,029 admissions from 2018–2023) and the MIMIC-IV database (299,712 admissions from 2008–2019). Access to both datasets was approved by the respective IRBs.  **Data selection and handling:** Sepsis patients were identified according to Sepsis-3 criteria. Only the first ICU admission was retained per patient to avoid duplication. Exclusion criteria applied to both cohorts included: age <18 years, ICU stay <48 hours, fewer than four glucose measurements within 48 hours of admission, unplanned discharge, or missing follow-up data. These ensured adequate temporal coverage for glucose trajectory modeling and reliable outcome assessment.  **Preprocessing:** Blood glucose values were extracted at 8-hour intervals during the first 48 hours and standardized using z-score transformation. Missing values within this time frame were imputed using the patient’s mean glucose level. All preprocessing procedures, including variable extraction and cleaning, were replicated identically in the external validation cohort. |
|  | M3 | **Was the data split into separate train, validation, and test sets?** Are there any differences in how test and training sets were selected and processed? How were patients or cases that occur more than once handled? Can they be found in both the test and training set? For example, same patient at different points in time or duplicate data. Are positive and negative cases from different sources? (For example, perhaps different machines are used in high- or low-probability settings, and the algorithm learns this pattern instead?) If there were minimum requirements on the data, state what those requirements were. | **Dataset splitting:** This study used two fully independent datasets, with FAH-SYSU serving as the derivation (training) cohort and MIMIC-IV as the external validation (test) cohort. There was no data overlap or shared patients between these cohorts.  **Repeated cases:** Only the first ICU admission per patient was included. No patient was duplicated or appeared in both cohorts.  **Positive/negative case sources:** All outcomes (e.g., 1-year mortality) were recorded within each cohort from the same source. There were no systematic differences in source or equipment between outcome groups.  **Minimum data requirements:** Inclusion required ≥4 blood glucose measurements in the first 48 hours post-ICU admission, and complete follow-up information. |
|  | M4 | **Specify if there was a human–AI interaction handling input data and level of expertise of the people handling it.** How was the ground truth established (e.g., double review with consensus, consensus review, single review, secondary sources)? What was the level of expertise of the source or reviewers? What level of noise was present (e.g., Cohen’s kappa).  If there was training involved in handling the data, this should be specified. | No human–AI interaction was involved in the input data acquisition or preprocessing stages. All data were structured and extracted using SQL queries from electronic medical records by trained clinical researchers and data scientists. One author (S. Li), certified in using the MIMIC-IV database, led the data extraction and quality checks.  The ground truth (e.g., sepsis diagnosis, 1-year mortality) was based on structured clinical criteria and confirmed EMR fields. Sepsis was defined using Sepsis-3 criteria algorithmically applied to diagnosis codes, infection suspicion indicators, and SOFA scores. Mortality outcomes were directly extracted from hospital or public records, requiring no subjective interpretation.  Given that ground truth labels were derived from validated structured data and established diagnostic definitions, there was no manual annotation or review requiring inter-rater reliability measures such as Cohen’s kappa.  All researchers involved in data handling had clinical backgrounds or were trained in critical care data analysis and EMR querying. |
|  | M5 | **Describe how missing or poor-quality data was handled.** Were extreme values or outliers handled separately? Explain how and why. | Missing blood glucose values within the first 48 hours after ICU admission were imputed using the individual patient’s mean of available measurements to preserve trajectory continuity for clustering. Patients with fewer than four glucose measurements during this period were excluded entirely to ensure data completeness and robustness of trajectory modeling.  No additional filtering or winsorization of extreme glucose values was performed, as these values may represent true clinical states (e.g., severe hyper- or hypoglycemia) relevant to sepsis prognosis. Other variables with missing data were handled through complete-case analysis where applicable. The quality of structured EMR data was assessed during preprocessing, and variables with systematic errors or missingness >20% were excluded from model development. |
|  | M6 | **State the AI model’s specifications, design, and the parameters used in training it.** The model’s data requirements, to serve its purpose, need to be clearly stated (e.g., data format, dimensions, time, etc.). How was the data preprocessed? It should be stated separately for training and test sets. What was the model architecture? Was a pre-trained model used? Was it pre-trained for the current study? If it was a pre-trained model, is the data the model was pre-trained on also part of the current data sets? What regularizers were used? (For example, dropout, white noise, batch normalization, stochastic weight averaging, etc.) How was the loss calculated? If a non-standard loss function was used, why was this particular loss chosen? What model-specific parameters were used in training the model? For example, learning rate, number of epochs, etc. | The AI model used was an unsupervised K-means clustering algorithm implemented via Scikit-learn (version 1.5.2). The goal was to identify distinct glycemic trajectory phenotypes based on maximum blood glucose values recorded every 8 hours within the first 48 hours after ICU admission. This resulted in 6 time points per patient.  **Data preprocessing:** Blood glucose values were standardized (z-score normalization) within each cohort. For patients with missing glucose readings at specific time points, the individual’s mean value across available time points was imputed. Patients with fewer than 4 glucose values in the first 48 hours were excluded. The same preprocessing pipeline was applied to both the derivation and external validation datasets.  **Model specifications:** The number of clusters (K) was selected based on multiple internal validation indices: Silhouette Score, Calinski-Harabasz index, Davies-Bouldin index, and Sum of Squared Errors (SSE), along with visual inspection using an elbow plot. No pre-trained model was used, and no external data overlapped with the study datasets.  As K-means is a deterministic optimization algorithm and not a neural model, no regularizers, loss functions, or parameters like learning rate or epochs were applicable. Instead, the model aimed to minimize within-cluster variance (inertia) to determine final cluster assignments. |
|  | M7 | **State the specific version of the AI model used in the study.** AI models are likely to undergo many iterations. It is important for reproducibility and tracking changes in the model if reused or implemented in a later study. | The AI model used in this study was the KMeans implementation from the Scikit-learn library, version 1.5.2. All analyses were conducted using Python (version 3.12.2). The model was not modified beyond standard parameter tuning (e.g., number of clusters). No proprietary or customized version of the algorithm was used, ensuring full reproducibility with the stated software versions. |
|  | M8 | **Specify the output of the AI. The output affects the model interpretation and post-processing.** What was the type of output? For example, probabilities, bounding boxes, text, segmented images, models? | The output of the AI model was cluster assignments (i.e., categorical labels indicating the group membership) for each patient based on their blood glucose trajectories within the first 48 hours of ICU admission. These outputs were used to stratify patients into clinically relevant subgroups for further comparison of outcomes and characteristics. No probabilistic or continuous outputs were generated by the model. |
|  | M9 | **Explain how the output contributed to decision-making and evaluation of the model.** In what way was the output decided upon? If the output was used in later steps, how was it used? | The output—cluster membership labels based on early glucose trajectory patterns—was used to stratify sepsis patients into distinct phenotypes. These groupings enabled subsequent statistical comparisons of baseline characteristics, organ support needs, and survival outcomes, including 1-year mortality. The number of clusters was determined through multiple internal validation metrics (SSE, Silhouette Score, Calinski-Harabasz, Davies-Bouldin) and visual inspection of the elbow plot. No decision threshold was involved, as the KMeans algorithm assigns each case to a single cluster deterministically. The outputs were not directly used for clinical decisions but informed downstream statistical analyses and subgroup interpretations. |
|  | M10 | **How was outcome performance measured?** The performance measure most likely familiar to the clinicians should be the primary reporting measure. Specify the exact version of the measure used. How was confidence evaluated? | The primary performance evaluation was based on 1-year all-cause mortality, a clinically familiar and relevant endpoint for ICU clinicians. Model output (cluster assignment) was evaluated by comparing survival across clusters using Kaplan–Meier survival curves and the log-rank test. Additionally, Cox proportional hazards regression models (univariate and multivariate) were used to estimate hazard ratios (HRs) and their 95% confidence intervals (CIs). Subgroup and sensitivity analyses were also performed to assess the consistency of these associations. Confidence intervals were derived using standard error estimates from the Cox model outputs. The choice of survival analysis metrics was driven by their interpretability and direct relevance to patient prognosis in sepsis. |
| RESULTS | R1 | **Describe the results of analysis and performance errors.** If no such analysis was performed, justify why not. Performance errors and failure analysis are important for AI models and help communicate the limitations of the model. | The study reported significant differences in clinical outcomes across the identified glycemic trajectory clusters, including 1-year mortality, 30-day mortality, and ICU length of stay. Performance was further validated using Kaplan–Meier survival analysis and multivariate Cox regression. Subgroup and sensitivity analyses confirmed the robustness of findings across age, gender, diabetic status, septic shock, and surgical status. However, a dedicated failure or error analysis (e.g., misclassification analysis or outcome deviation by subgroup) was not conducted. This was because the study employed an unsupervised clustering approach, not a supervised prediction model, and thus did not generate prediction errors per case. The model’s utility lies in stratifying patients based on glycemic dynamics rather than individual risk prediction. Limitations of this approach are acknowledged in the discussion. |
| DISCUSSION AND OTHER INFORMATION | D1 | **State if and how the AI model/data can be accessed, including any restrictions to access or reuse.** If it is not possible, state why. Include any details and license. | The AI model and data used in this study are based on two publicly available databases: the First Affiliated Hospital of Sun Yat-sen University (FAH-SYSU) database and the Medical Information Mart for Intensive Care IV (MIMIC-IV) database. Access to the MIMIC-IV dataset is available through the PhysioNet platform under the required data use agreement. The FAH-SYSU dataset is restricted to authorized researchers and requires institutional access. As the data were de-identified and fully anonymized, there are no personal identifiers involved in the datasets. The datasets used and analyzed during the current study are available from the corresponding author upon reasonable request. The model itself, based on unsupervised clustering, is not distributed due to the lack of a pre-trained version. |
|  | D2 | **Describe ethical considerations and implications of the model, and/or research.** Biases and limitations, the input data or output, that impact generalizability should also be considered. | Ethical approval for this study was obtained from the Clinical Research Ethics Committee of the First Affiliated Hospital of Sun Yat-sen University and the Beth Israel Deaconess Medical Center, with a waiver of informed consent granted for the use of de-identified MIMIC-IV data. Despite the ethical approvals, there are inherent biases in the input data: the data from the FAH-SYSU cohort are geographically specific to China, and the MIMIC-IV cohort represents data from a U.S.-based hospital. These biases may limit the generalizability of the model across diverse populations or healthcare settings. Furthermore, the unsupervised clustering method may miss important patient subgroups that could benefit from more detailed supervised prediction models. Finally, while glycemic control trajectories were used as features, other unmeasured variables, such as healthcare practices and interventions, may have influenced patient outcomes. |
